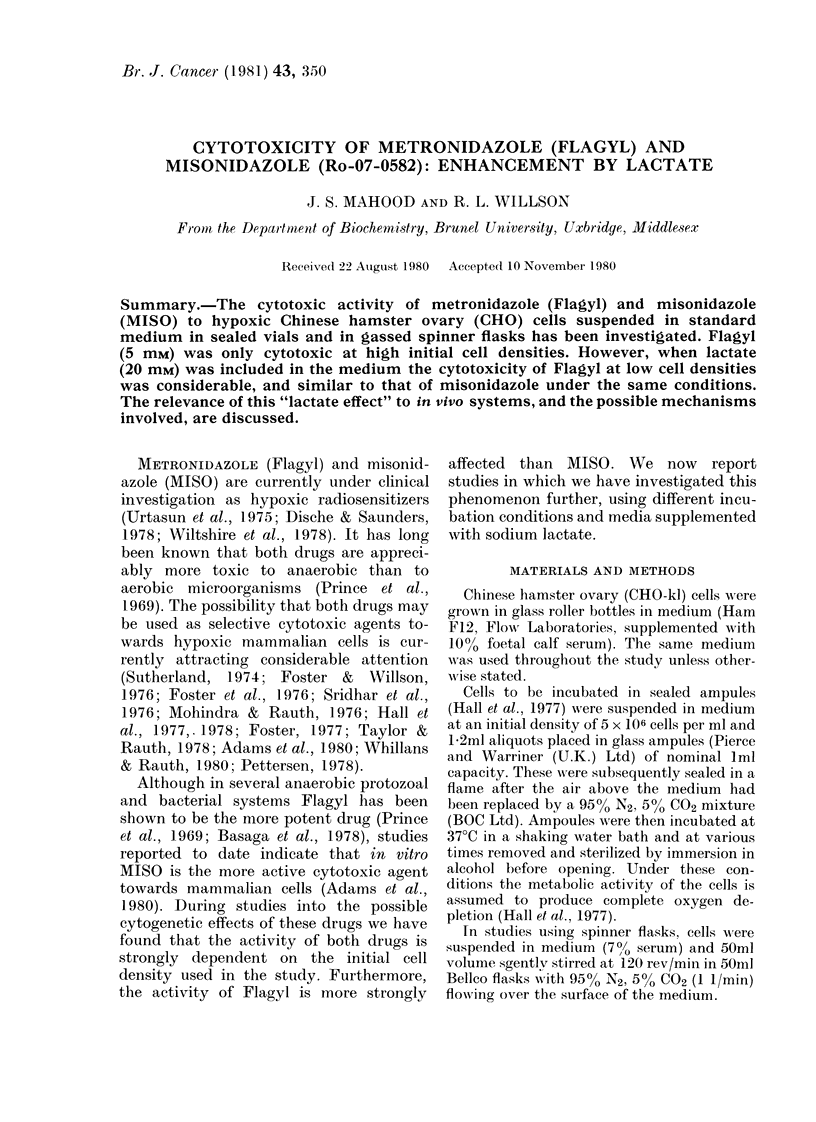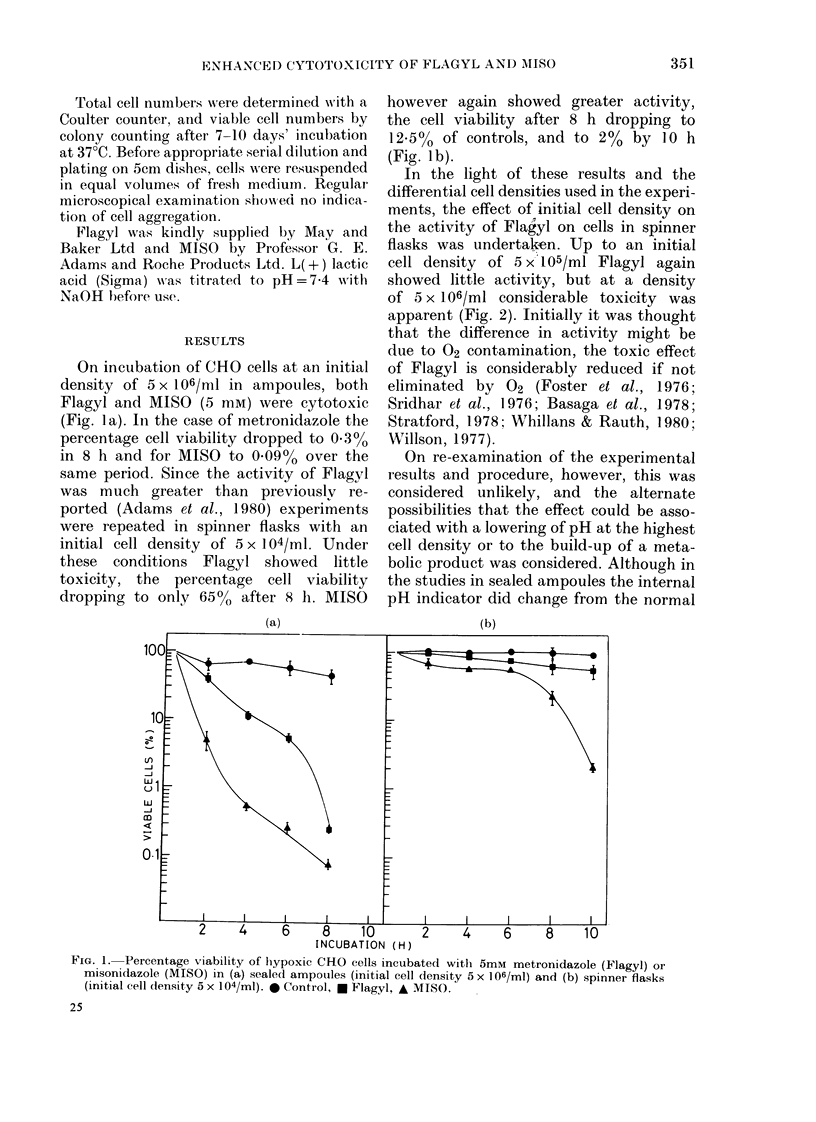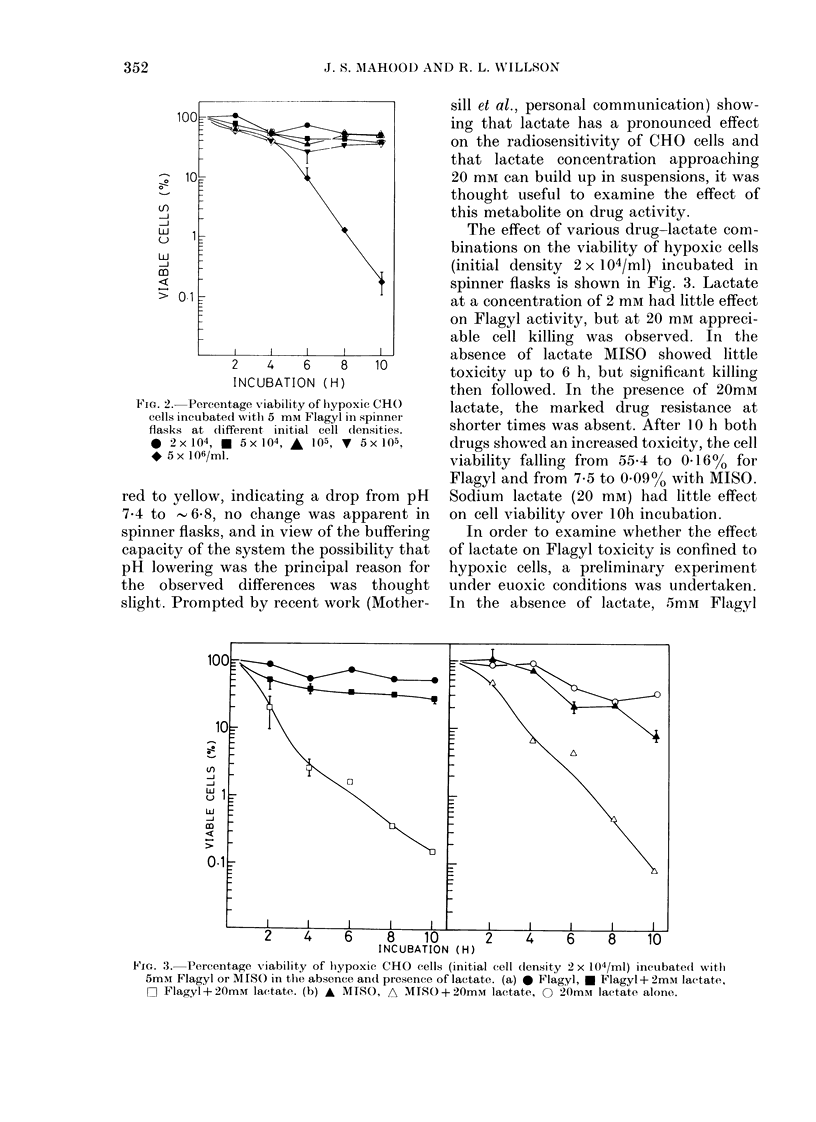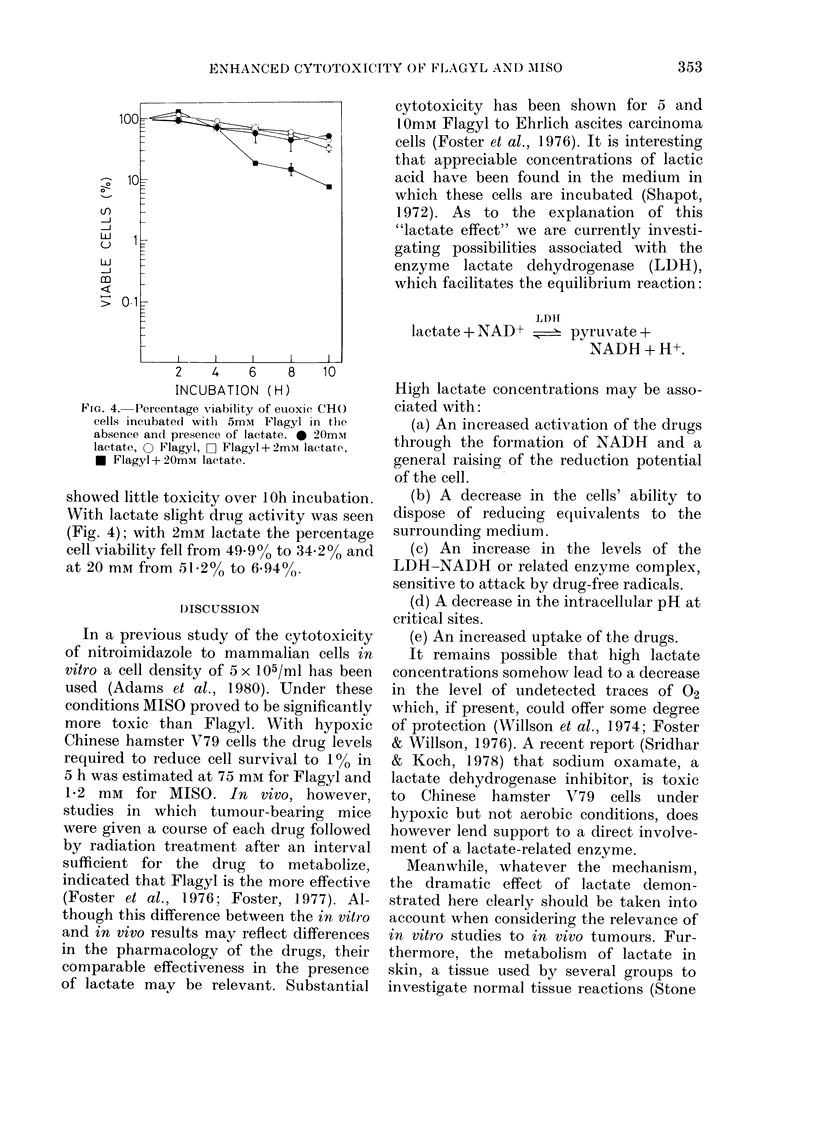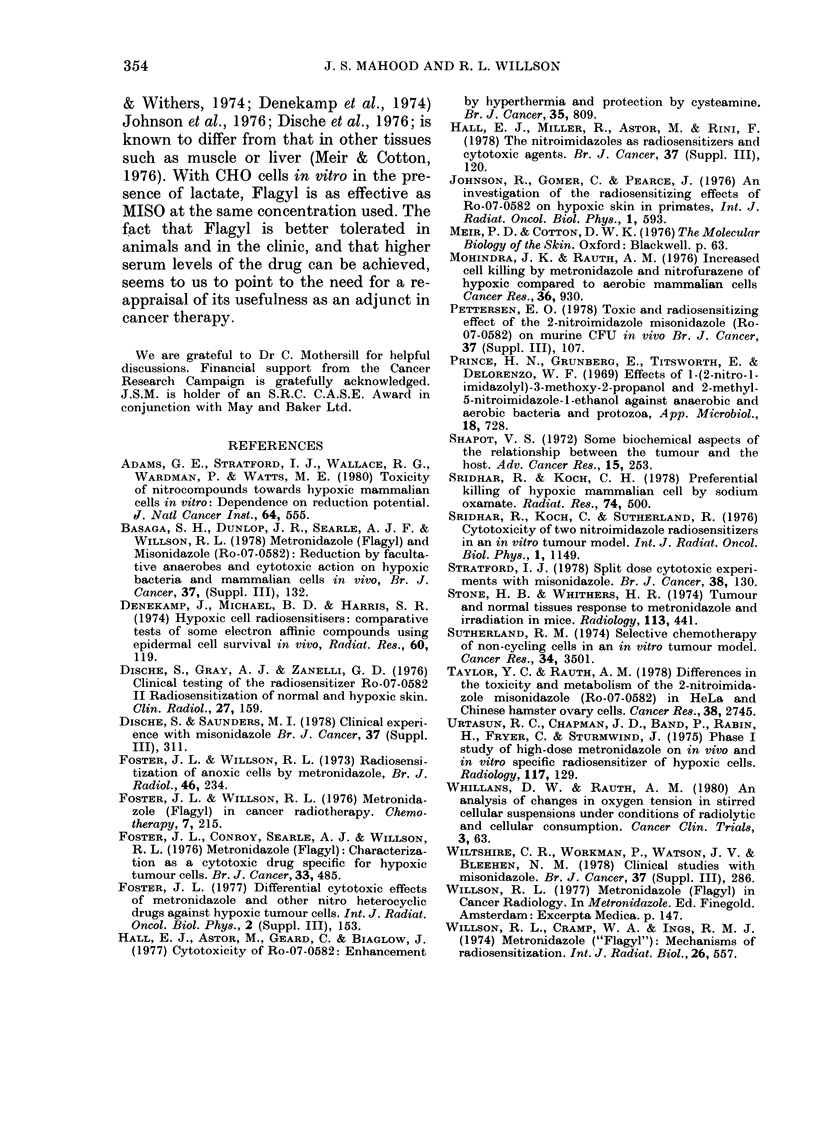# Cytotoxicity of metronidazole (Flagyl) and misonidazole (Ro-07-0582): enhancement by lactate.

**DOI:** 10.1038/bjc.1981.55

**Published:** 1981-03

**Authors:** J. S. Mahood, R. L. Willson

## Abstract

The cytotoxic activity of metronidazole (Flagyl) and misonidazole (MISO) to hypoxic Chinese hamster ovary (CHO) cells suspended in standard medium in sealed vials and in gassed spinner flasks has been investigated. Flagyl (5 mM) was only cytotoxic at high initial cell densities. However, when lactate (20 mM) was included in the medium the cytotoxicity of Flagyl at low cell densities was considerable, and similar to that of misonidazole under the same conditions. The relevance of this "lactate effect" to in vivo systems, and the possible mechanisms involved, are discussed.


*Br. J. Cancer* (1981) **43**, 350

### CYTOTOXICITY OF METRONIDAZOLE (FLAGYL) AND MISONIDAZOLE (Ro-07-0582): ENHANCEMENT BY LACTATE

J. S. MAHOOD AND R. L. WILLSON

*From the Department of Biochemistry, Brunel University, Uxbridge, Middlesex*

Received 22 August 1980 Accepted 10 November 1980

**Summary.—The cytotoxic activity of metronidazole (Flagyl) and misonidazole (MISO) to hypoxic Chinese hamster ovary (CHO) cells suspended in standard medium in sealed vials and in gassed spinner flasks has been investigated. Flagyl (5 mM) was only cytotoxic at high initial cell densities. However, when lactate (20 mM) was included in the medium the cytotoxicity of Flagyl at low cell densities was considerable, and similar to that of misonidazole under the same conditions. The relevance of this "lactate effect" to *in vivo* systems, and the possible mechanisms involved, are discussed.**

METRONIDAZOLE (Flagyl) and misonidazole (MISO) are currently under clinical investigation as hypoxic radiosensitizers (Urtasun *et al.*, 1975; Dische & Saunders, 1978; Wiltshire *et al.*, 1978). It has long been known that both drugs are appreciably more toxic to anaerobic than to aerobic microorganisms (Prince *et al.*, 1969). The possibility that both drugs may be used as selective cytotoxic agents towards hypoxic mammalian cells is currently attracting considerable attention (Sutherland, 1974; Foster & Willson, 1976; Foster *et al.*, 1976; Sridhar *et al.*, 1976; Mohindra & Rauth, 1976; Hall *et al.*, 1977, 1978; Foster, 1977; Taylor & Rauth, 1978; Adams *et al.*, 1980; Whillans & Rauth, 1980; Pettersen, 1978).

Although in several anaerobic protozoal and bacterial systems Flagyl has been shown to be the more potent drug (Prince *et al.*, 1969; Basaga *et al.*, 1978), studies reported to date indicate that *in vitro* MISO is the more active cytotoxic agent towards mammalian cells (Adams *et al.*, 1980). During studies into the possible cytogenetic effects of these drugs we have found that the activity of both drugs is strongly dependent on the initial cell density used in the study. Furthermore, the activity of Flagyl is more strongly affected than MISO. We now report studies in which we have investigated this phenomenon further, using different incubation conditions and media supplemented with sodium lactate.

### MATERIALS AND METHODS

Chinese hamster ovary (CHO-kl) cells were grown in glass roller bottles in medium (Ham F12, Flow Laboratories, supplemented with 10% foetal calf serum). The same medium was used throughout the study unless otherwise stated.

Cells to be incubated in sealed ampules (Hall *et al.*, 1977) were suspended in medium at an initial density of $5 \times 10^6$ cells per ml and 1·2ml aliquots placed in glass ampules (Pierce and Warriner (U.K.) Ltd) of nominal 1ml capacity. These were subsequently sealed in a flame after the air above the medium had been replaced by a 95% $N_2$, 5% $CO_2$ mixture (BOC Ltd). Ampoules were then incubated at 37°C in a shaking water bath and at various times removed and sterilized by immersion in alcohol before opening. Under these conditions the metabolic activity of the cells is assumed to produce complete oxygen depletion (Hall *et al.*, 1977).

In studies using spinner flasks, cells were suspended in medium (7% serum) and 50ml volume sgently stirred at 120 rev/min in 50ml Bellco flasks with 95% $N_2$, 5% $CO_2$ (1 l/min) flowing over the surface of the medium.

Total cell numbers were determined with a Coulter counter, and viable cell numbers by colony counting after 7–10 days' incubation at 37°C. Before appropriate serial dilution and plating on 5cm dishes, cells were resuspended in equal volumes of fresh medium. Regular microscopical examination showed no indication of cell aggregation.

Flagyl was kindly supplied by May and Baker Ltd and MISO by Professor G. E. Adams and Roche Products Ltd. L(+) lactic acid (Sigma) was titrated to pH = 7·4 with NaOH before use.

### RESULTS

On incubation of CHO cells at an initial density of $5 \times 10^6$/ml in ampoules, both Flagyl and MISO (5 mM) were cytotoxic (Fig. 1a). In the case of metronidazole the percentage cell viability dropped to 0·3% in 8 h and for MISO to 0·09% over the same period. Since the activity of Flagyl was much greater than previously reported (Adams *et al.*, 1980) experiments were repeated in spinner flasks with an initial cell density of $5 \times 10^4$/ml. Under these conditions Flagyl showed little toxicity, the percentage cell viability dropping to only 65% after 8 h. MISO however again showed greater activity, the cell viability after 8 h dropping to 12·5% of controls, and to 2% by 10 h (Fig. 1b).

In the light of these results and the differential cell densities used in the experiments, the effect of initial cell density on the activity of Flagyl on cells in spinner flasks was undertaken. Up to an initial cell density of $5 \times 10^5$/ml Flagyl again showed little activity, but at a density of $5 \times 10^6$/ml considerable toxicity was apparent (Fig. 2). Initially it was thought that the difference in activity might be due to $O_2$ contamination, the toxic effect of Flagyl is considerably reduced if not eliminated by $O_2$ (Foster *et al.*, 1976; Sridhar *et al.*, 1976; Basaga *et al.*, 1978; Stratford, 1978; Whillans & Rauth, 1980; Willson, 1977).

On re-examination of the experimental results and procedure, however, this was considered unlikely, and the alternate possibilities that the effect could be associated with a lowering of pH at the highest cell density or to the build-up of a metabolic product was considered. Although in the studies in sealed ampoules the internal pH indicator did change from the normal

FIG. 1.—Percentage viability of hypoxic CHO cells incubated with 5mM metronidazole (Flagyl) or misonidazole (MISO) in (a) sealed ampoules (initial cell density $5 \times 10^6$/ml) and (b) spinner flasks (initial cell density $5 \times 10^4$/ml). ● Control, ■ Flagyl, ▲ MISO.

FIG. 2.—Percentage viability of hypoxic CHO cells incubated with 5 mM Flagyl in spinner flasks at different initial cell densities. ● $2 \times 10^4$, ■ $5 \times 10^4$, ▲ $10^5$, ▼ $5 \times 10^5$, ◆ $5 \times 10^6$/ml.

red to yellow, indicating a drop from pH 7·4 to ~6·8, no change was apparent in spinner flasks, and in view of the buffering capacity of the system the possibility that pH lowering was the principal reason for the observed differences was thought slight. Prompted by recent work (Mothersill *et al.*, personal communication) showing that lactate has a pronounced effect on the radiosensitivity of CHO cells and that lactate concentration approaching 20 mM can build up in suspensions, it was thought useful to examine the effect of this metabolite on drug activity.

The effect of various drug–lactate combinations on the viability of hypoxic cells (initial density $2 \times 10^4$/ml) incubated in spinner flasks is shown in Fig. 3. Lactate at a concentration of 2 mM had little effect on Flagyl activity, but at 20 mM appreciable cell killing was observed. In the absence of lactate MISO showed little toxicity up to 6 h, but significant killing then followed. In the presence of 20mM lactate, the marked drug resistance at shorter times was absent. After 10 h both drugs showed an increased toxicity, the cell viability falling from 55·4 to 0·16% for Flagyl and from 7·5 to 0·09% with MISO. Sodium lactate (20 mM) had little effect on cell viability over 10h incubation.

In order to examine whether the effect of lactate on Flagyl toxicity is confined to hypoxic cells, a preliminary experiment under euoxic conditions was undertaken. In the absence of lactate, 5mM Flagyl

FIG. 3.—Percentage viability of hypoxic CHO cells (initial cell density $2 \times 10^4$/ml) incubated with 5mM Flagyl or MISO in the absence and presence of lactate. (a) ● Flagyl, ■ Flagyl + 2mM lactate, □ Flagyl + 20mM lactate. (b) ▲ MISO, △ MISO + 20mM lactate, ○ 20mM lactate alone.

FIG. 4.—Percentage viability of euoxic CHO cells incubated with 5mM Flagyl in the absence and presence of lactate. ● 20mM lactate, ○ Flagyl, □ Flagyl + 2mM lactate, ■ Flagyl + 20mM lactate.

showed little toxicity over 10h incubation. With lactate slight drug activity was seen (Fig. 4); with 2mM lactate the percentage cell viability fell from 49·9% to 34·2% and at 20 mM from 51·2% to 6·94%.

### DISCUSSION

In a previous study of the cytotoxicity of nitroimidazole to mammalian cells *in vitro* a cell density of $5 \times 10^5$/ml has been used (Adams *et al.*, 1980). Under these conditions MISO proved to be significantly more toxic than Flagyl. With hypoxic Chinese hamster V79 cells the drug levels required to reduce cell survival to 1% in 5 h was estimated at 75 mM for Flagyl and 1·2 mM for MISO. *In vivo*, however, studies in which tumour-bearing mice were given a course of each drug followed by radiation treatment after an interval sufficient for the drug to metabolize, indicated that Flagyl is the more effective (Foster *et al.*, 1976; Foster, 1977). Although this difference between the *in vitro* and *in vivo* results may reflect differences in the pharmacology of the drugs, their comparable effectiveness in the presence of lactate may be relevant. Substantial cytotoxicity has been shown for 5 and 10mM Flagyl to Ehrlich ascites carcinoma cells (Foster *et al.*, 1976). It is interesting that appreciable concentrations of lactic acid have been found in the medium in which these cells are incubated (Shapot, 1972). As to the explanation of this "lactate effect" we are currently investigating possibilities associated with the enzyme lactate dehydrogenase (LDH), which facilitates the equilibrium reaction:

$$\text{lactate} + \text{NAD}^+ \underset{}{\overset{\text{LDH}}{\rightleftharpoons}} \text{pyruvate} + \text{NADH} + \text{H}^+.$$

High lactate concentrations may be associated with:

(a) An increased activation of the drugs through the formation of NADH and a general raising of the reduction potential of the cell.

(b) A decrease in the cells' ability to dispose of reducing equivalents to the surrounding medium.

(c) An increase in the levels of the LDH–NADH or related enzyme complex, sensitive to attack by drug-free radicals.

(d) A decrease in the intracellular pH at critical sites.

(e) An increased uptake of the drugs.

It remains possible that high lactate concentrations somehow lead to a decrease in the level of undetected traces of $O_2$ which, if present, could offer some degree of protection (Willson *et al.*, 1974; Foster & Willson, 1976). A recent report (Sridhar & Koch, 1978) that sodium oxamate, a lactate dehydrogenase inhibitor, is toxic to Chinese hamster V79 cells under hypoxic but not aerobic conditions, does however lend support to a direct involvement of a lactate-related enzyme.

Meanwhile, whatever the mechanism, the dramatic effect of lactate demonstrated here clearly should be taken into account when considering the relevance of *in vitro* studies to *in vivo* tumours. Furthermore, the metabolism of lactate in skin, a tissue used by several groups to investigate normal tissue reactions (Stone

& Withers, 1974; Denekamp *et al.*, 1974) Johnson *et al.*, 1976; Dische *et al.*, 1976; is known to differ from that in other tissues such as muscle or liver (Meir & Cotton, 1976). With CHO cells *in vitro* in the presence of lactate, Flagyl is as effective as MISO at the same concentration used. The fact that Flagyl is better tolerated in animals and in the clinic, and that higher serum levels of the drug can be achieved, seems to us to point to the need for a reappraisal of its usefulness as an adjunct in cancer therapy.

We are grateful to Dr C. Mothersill for helpful discussions. Financial support from the Cancer Research Campaign is gratefully acknowledged. J.S.M. is holder of an S.R.C. C.A.S.E. Award in conjunction with May and Baker Ltd.

### REFERENCES

ADAMS, G. E., STRATFORD, I. J., WALLACE, R. G., WARDMAN, P. & WATTS, M. E. (1980) Toxicity of nitrocompounds towards hypoxic mammalian cells *in vitro*: Dependence on reduction potential. *J. Natl Cancer Inst.*, **64**, 555.

BASAGA, S. H., DUNLOP, J. R., SEARLE, A. J. F. & WILLSON, R. L. (1978) Metronidazole (Flagyl) and Misonidazole (Ro-07-0582): Reduction by facultative anaerobes and cytotoxic action on hypoxic bacteria and mammalian cells *in vivo*, *Br. J. Cancer*, **37**, (Suppl. III), 132.

DENEKAMP, J., MICHAEL, B. D. & HARRIS, S. R. (1974) Hypoxic cell radiosensitisers: comparative tests of some electron affinic compounds using epidermal cell survival *in vivo*, *Radiat. Res.*, **60**, 119.

DISCHE, S., GRAY, A. J. & ZANELLI, G. D. (1976) Clinical testing of the radiosensitizer Ro-07-0582 II Radiosensitization of normal and hypoxic skin. *Clin. Radiol.*, **27**, 159.

DISCHE, S. & SAUNDERS, M. I. (1978) Clinical experience with misonidazole *Br. J. Cancer*, **37** (Suppl. III), 311.

FOSTER, J. L. & WILLSON, R. L. (1973) Radiosensitization of anoxic cells by metronidazole, *Br. J. Radiol.*, **46**, 234.

FOSTER, J. L. & WILLSON, R. L. (1976) Metronidazole (Flagyl) in cancer radiotherapy. *Chemotherapy*, **7**, 215.

FOSTER, J. L., CONROY, SEARLE, A. J. & WILLSON, R. L. (1976) Metronidazole (Flagyl): Characterization as a cytotoxic drug specific for hypoxic tumour cells. *Br. J. Cancer*, **33**, 485.

FOSTER, J. L. (1977) Differential cytotoxic effects of metronidazole and other nitro heterocyclic drugs against hypoxic tumour cells. *Int. J. Radiat. Oncol. Biol. Phys.*, **2** (Suppl. III), 153.

HALL, E. J., ASTOR, M., GEARD, C. & BIAGLOW, J. (1977) Cytotoxicity of Ro-07-0582: Enhancement by hyperthermia and protection by cysteamine. *Br. J. Cancer*, **35**, 809.

HALL, E. J., MILLER, R., ASTOR, M. & RINI, F. (1978) The nitroimidazoles as radiosensitizers and cytotoxic agents. *Br. J. Cancer*, **37** (Suppl. III), 120.

JOHNSON, R., GOMER, C. & PEARCE, J. (1976) An investigation of the radiosensitizing effects of Ro-07-0582 on hypoxic skin in primates, *Int. J. Radiat. Oncol. Biol. Phys.*, **1**, 593.

MEIR, P. D. & COTTON, D. W. K. (1976) *The Molecular Biology of the Skin.* Oxford: Blackwell. p. 63.

MOHINDRA, J. K. & RAUTH, A. M. (1976) Increased cell killing by metronidazole and nitrofurazene of hypoxic compared to aerobic mammalian cells *Cancer Res.*, **36**, 930.

PETTERSEN, E. O. (1978) Toxic and radiosensitizing effect of the 2-nitroimidazole misonidazole (Ro-07-0582) on murine CFU *in vivo* *Br. J. Cancer*, **37** (Suppl. III), 107.

PRINCE, H. N., GRUNBERG, E., TITSWORTH, E. & DELORENZO, W. F. (1969) Effects of 1-(2-nitro-1-imidazolyl)-3-methoxy-2-propanol and 2-methyl-5-nitroimidazole-1-ethanol against anaerobic and aerobic bacteria and protozoa, *App. Microbiol.*, **18**, 728.

SHAPOT, V. S. (1972) Some biochemical aspects of the relationship between the tumour and the host. *Adv. Cancer Res.*, **15**, 253.

SRIDHAR, R. & KOCH, C. H. (1978) Preferential killing of hypoxic mammalian cell by sodium oxamate. *Radiat. Res.*, **74**, 500.

SRIDHAR, R., KOCH, C. & SUTHERLAND, R. (1976) Cytotoxicity of two nitroimidazole radiosensitizers in an *in vitro* tumour model. *Int. J. Radiat. Oncol. Biol. Phys.*, **1**, 1149.

STRATFORD, I. J. (1978) Split dose cytotoxic experiments with misonidazole. *Br. J. Cancer*, **38**, 130.

STONE, H. B. & WHITHERS, H. R. (1974) Tumour and normal tissues response to metronidazole and irradiation in mice. *Radiology*, **113**, 441.

SUTHERLAND, R. M. (1974) Selective chemotherapy of non-cycling cells in an *in vitro* tumour model. *Cancer Res.*, **34**, 3501.

TAYLOR, Y. C. & RAUTH, A. M. (1978) Differences in the toxicity and metabolism of the 2-nitroimidazole misonidazole (Ro-07-0582) in HeLa and Chinese hamster ovary cells. *Cancer Res.*, **38**, 2745.

URTASUN, R. C., CHAPMAN, J. D., BAND, P., RABIN, H., FRYER, C. & STURMWIND, J. (1975) Phase I study of high-dose metronidazole on *in vivo* and *in vitro* specific radiosensitizer of hypoxic cells. *Radiology*, **117**, 129.

WHILLANS, D. W. & RAUTH, A. M. (1980) An analysis of changes in oxygen tension in stirred cellular suspensions under conditions of radiolytic and cellular consumption. *Cancer Clin. Trials*, **3**, 63.

WILTSHIRE, C. R., WORKMAN, P., WATSON, J. V. & BLEEHEN, N. M. (1978) Clinical studies with misonidazole. *Br. J. Cancer*, **37** (Suppl. III), 286.

WILLSON, R. L. (1977) Metronidazole (Flagyl) in Cancer Radiology. In *Metronidazole*. Ed. Finegold. Amsterdam: Excerpta Medica. p. 147.

WILLSON, R. L., CRAMP, W. A. & INGS, R. M. J. (1974) Metronidazole ("Flagyl"): Mechanisms of radiosensitization. *Int. J. Radiat. Biol.*, **26**, 557.